# Editorial: Oral health and quality of life in vulnerable populations

**DOI:** 10.3389/froh.2025.1581194

**Published:** 2025-05-13

**Authors:** Vini Mehta, Mohmed Isaqali Karobari, Luca Fiorillo

**Affiliations:** ^1^ Department of Dental Cell Research, Dr. D.Y. Patil Dental College and Hospital, Dr. D.Y. Patil Vidyapeeth, Pune, India; ^2^ Department of Restorative Dentistry & Endodontics, Faculty of Dentistry, University of Puthisastra, Phnom Penh, Cambodia; ^3^ Conservative Dentistry & Endodontics, Saveetha Dental College & Hospitals, Saveetha Institute of Medical and Technical Sciences University, Chennai, India; ^4^ Conservative Dentistry Unit, School of Dental Sciences, Universiti Sains Malaysia, Kota Bharu, Malaysia; ^5^ Department of Dentistry, Faculty of Dental Sciences, University of Aldent, Tirana, Albania; ^6^ Department of Biomedical and Dental Sciences, Morphological and Functional Images, University of Messina, Messina, Italy; ^7^ Multidisciplinary Department of Medical-Surgical and Odontostomatological Specialties, University of Campania“Luigi Vanvitelli”, Naples, Italy

**Keywords:** oral health disparities, vulnerable populations, quality of life, health equity, oral health promotion

**Editorial on the Research Topic**
Oral health and quality of life in vulnerable populations

Oral health is crucial for overall well-being. While everyone is at risk of oral health problems, some individuals, groups, or communities bear greater risk due to socioeconomic determinants of health that influence their lives and lifestyle practices. Vulnerable groups may include women, children, the elderly, special needs population, people involved with justice system, the LGBTQ+ community, refugees, slum dwellers, racial and ethnic minorities, and those living in underserved areas. Vulnerable populations commonly face obstacles that restrict their access (1, 2) to quality dental care, leading to higher rates of oral diseases. These barriers encompass socioeconomic challenges, cultural and linguistic differences, and systemic inequities in healthcare delivery (3). The COVID-19 pandemic has intensified these disparities, underscoring the urgent need for targeted interventions to promote oral health equity (4). This editorial outline the research objectives and emphasizes efforts to enhance oral health care for marginalized community groups.

The significance of oral health education and promotion is a recurring theme in the papers featured in this special issue. Numerous studies emphasize the relevance of culturally relevant educational initiatives that empower individuals with understanding of oral hygiene practices, preventive care, and the significance of regular dental appointments. By fostering a deeper understanding of oral health and including community members to develop contextually relevant materials, these initiatives can improve health outcomes and reduce burden among vulnerable groups (5, 6).

This special issue also explores innovative strategies such as Mobile dental clinics, community-based interventions, and telehealth solutions to enhance oral health care access for underserved populations. These approaches help overcome common barriers such as transportation issues, fear of dental treatment, and financial constraints. However, it is imperative to also consider the challenges associated with implementation of these innovative strategies. For instance, tele dentistry are fraught with challenges related to lack of appropriate devices resulting in low quality of images required for proper diagnostic evaluation a, low financial returns for the participating dentist, lack of trained staffs, lack of good internet connectivity, hardware and software support, and patients' preference to face-to-face consultation over the virtual consultation (7). Similarly, although mobile dentistry clinics offer services on-the-go, the primary challenges are the time required to commute and set up the clinic, high insurance cost for the driver and the vehicle, the requirement for skilled personnel to handle technical and mechanical issues, and lack of availability for patient follow-up and complication management (8).

Another crucial aspect examined is the intersection between social determinants of health and oral health outcomes. Social capital, defined as the resources accessed through social networks and trust, plays an important role in shaping oral health behaviors and results. Studies in this issue explore how factors such as income, education, and social support affect oral health status and access to care, highlighting the need for comprehensive, multidisciplinary strategies to address oral health disparities (9, 10). However, there is lack of primary studies which have explored the relationship of social capital with oral diseases. Quality of life is another central theme across the contributions, as oral health significantly affects an individual's ability to eat, speak, and engage socially. The articles demonstrate that poor oral health can lead to pain, discomfort, and functional limitations, ultimately impacting mental health, self-esteem, and overall quality of life. Therefore, addressing oral health disparities is not just a matter of dental care, but also a crucial step in promoting social justice and enhancing population health (Gudsoorkar et al., 11).

This special issue also examines the role of collaborative care models involving oral health professionals and other healthcare providers. Integrated care models that incorporate oral health into primary care settings, community health programs, and interdisciplinary healthcare teams can help bridge care gaps for vulnerable populations. Such models have the potential to improve early oral diseases detection, facilitate timely interventions, and enhance overall health and well-being of marginalized individuals (12, Mehta et al.).

Looking forward, the future of oral health care for vulnerable populations will increasingly integrate artificial intelligence (AI) to enhance accessibility, efficiency, and personalization. AI-powered diagnostic tools will enable early detection of oral diseases through automated image analysis, reducing the burden of advanced dental conditions. AI-driven tele-dentistry solutions will expand access to care in remote and underserved communities, offering virtual consultations and treatment planning. Machine learning algorithms will analyze large datasets to predict oral health risks and optimize preventive strategies tailored to individual needs (13–15). AI-driven chatbots and virtual assistants will support patient education, behavioral coaching, and adherence to oral hygiene practices, ultimately improving health outcomes and reducing disparities.

In conclusion, this special issue provides nuanced insights into the challenges and opportunities in advancing oral health care of vulnerable populations. To achieve oral health equity, policymakers and healthcare professionals must take decisive actions such as increasing financial incentives for health professionals working in underserved regions, integrating oral health into primary healthcare services, and tackling underlying socioeconomic determinants of oral health to address oral health disparities. Overall, the research papers published in this special issue calls for further research on developing sustainable, multimodal, evidence-based, and tailored interventions to achieve oral health equity for all.
